# Volatile Organic Compounds from Entomopathogenic and Nematophagous Fungi, Repel Banana Black Weevil (*Cosmopolites sordidus*)

**DOI:** 10.3390/insects11080509

**Published:** 2020-08-06

**Authors:** Ana Lozano-Soria, Ugo Picciotti, Federico Lopez-Moya, Javier Lopez-Cepero, Francesco Porcelli, Luis Vicente Lopez-Llorca

**Affiliations:** 1 Department of Marine Science and Applied Biology, Laboratory of Plant Pathology, University of Alicante, 03080 Alicante, Spain; federico.lopez@ua.es (F.L.-M.); lv.lopez@ua.es (L.V.L.-L.); 2 Department of Soil, Plant, and Food Sciences—(DiSSPA), University of Bari Aldo Moro, 70126 Bari, Italy; francesco.porcelli@uniba.it; 3 Dyrecta Lab Istituto di Ricerca, 70014 Conversano, Bari, Italy; 4 Technical Department of Coplaca, 38001 Tenerife, Spain; cepero@coplaca.org

**Keywords:** Curculionidae, *Musa accuminata*, invasive species, pest control, biological control, VOCs, olfactory response, semiochemicals, pheromone, *Cosmopolites sordidus*

## Abstract

**Simple Summary:**

Banana cultivation is crucial in all the tropical countries of the world. Banana weevil (*Cosmopolites sordidus*) is a coleoptera whose larvae feed on the corm of the plant making tunnels on the inside. Tunneling causes reductions in crop yield due to the loss of plants, by decaying or breakage of rhizomes and reduces the weight of the bunches. Banana weevil in Spain is present in almost all the islands of the Canary archipelago, so it is important to control this pest. This control is carried out by means of preventive or natural techniques, removing infected banana plants or by using traps with aggregation pheromones or kairomones. A sustainable alternative would be the use of biological control agents isolated and evaluated beforehand. To this end, laboratory tests have been carried out on the study of the behavior of the banana weevil with volatile compounds naturally present in fungi isolated from the banana crops of Tenerife. Laboratory tests indicated that the compounds repel banana weevil and mask the attractive activity of kairomone. The implementation of the results for the management of the pest could produce advancements in the agrobiotechnological sustainability of the world banana cultivation, increasing its production.

**Abstract:**

Fungal Volatile Organic Compounds (VOCs) repel banana black weevil (BW), *Cosmopolites sordidus* (Germar, 1824), the key-pest of banana [*Musa* sp. (Linnaeus, 1753)]. The entomopathogens *Beauveria bassiana* (Bb1TS11) and *Metarhizium robertsii* (Mr4TS04) were isolated from banana plantation soils using an insect bait. Bb1TS11 and Mr4TS04 were pathogenic to BW adults. Bb1TS11, Bb203 (from infected palm weevils), Mr4TS04 and the nematophagous fungus *Pochonia clamydosporia* (Pc123), were tested for VOCs production. VOCs were identified by Gas Chromatography/Mass Spectrometry–Solid-Phase Micro Extraction (GC/MS-SPME). GC/MS-SPME identified a total of 97 VOCs in all strains tested. Seven VOCs (styrene, benzothiazole, camphor, borneol, 1,3-dimethoxy-benzene, 1-octen-3-ol and 3-cyclohepten-1-one) were selected for their abundance or previous record as insect repellents. In olfactometry bioassays, BW-starved adults in the dark showed the highest mobility to banana corm. 2-cyclohepten-1-one (C7), commercially available isomer of 3-cyclohepten-1-one, is the best BW repellent (*p* < 0.05), followed by 1,3-dimethoxy-benzene (C5). The rest of the VOCs have a milder repellency to BW. Styrene (C1) and benzothiazole (C2) (known to repel palm weevil) block the attraction of banana corm and BW pheromone to BW adults in bioassays. Therefore, VOCs from biocontrol fungi can be used in future studies to biomanage BW in the field.

## 1. Introduction

Banana black weevil (BW), *Cosmopolites sordidus* (Germar, 1824; Coleoptera: Curculionidae) is the key pest of banana plant crops [1,2,3,4]. BW causes more damage to banana crops than any other arthropod pest. BW infestations alter nutrient and water uptakes, causing a decline in plant vigor, size and toppling [3,4,5]. *C. sordidus* originates from South-East Asia [6] and has been invading Central Africa, Central America, the Pacific Islands and all regions where bananas are cultivated between 30°N and 31°S latitudes [7]. In Europe, BW is present in Madeira (Portugal) [8] and the Canary Islands (Spain) [9]. *C. sordidus* can cause losses from 30% up to 90% of the total banana crop yield production in intense outbreaks [10,11,12].

BW adults are active at night and prefer a humid environment, thriving in crop residues. They are slow-moving and poor flyers despite its functional wings. Therefore, the pest dispersion is mainly passive occurring through the handling of infested plant material. Females have low fertility, laying 1–4 eggs/week with considerable intervals during their adult life and, reaching 10–270 total eggs in their lifetime [13]. BW larvae go through 5–8 stages and the post-embryonic development is completed within 5–7 weeks under tropical conditions [13]. BW larvae damage the banana plants, boring the corm for feeding [14]. BW tunnels reduce nutrient absorption and can weaken the plant. This leads to reductions in fruit production or falling of bunches [13]. In massive infestations, plants can rot and die.

Banana weevils have several predators and pathogens. BW pathogens include entomopathogenic fungi (*Metarhizium anisopliae* and *Beauveria bassiana*) and nematodes. *B. bassiana* [(Balsamo) Vuillemin, 1912] and *M. anisopliae* [(Metschnikoff) Sorokin, 1883] of the Hypocreales (Ascomycota) have been used for the biological control of important agricultural pests [15,16,17,18]. These fungi have potential for *C. sordidus* biocontrol under laboratory [19] and field [5] conditions.

*Pochonia chlamydosporia* [(Goddard) Zare and Gams, 2001] is a nematophagous fungus very close phylogenomically to *M. anisopliae* [20,21]. *P. chlamydosporia* parasitizes nematode eggs and females [22,23,24], it is also an endophyte [25,26] and a soil fungus [27,28]. Therefore, it has been used as a biocontrol agent of cyst and root knot nematodes [29].

Volatile organic compounds (VOCs) are solid and liquid carbon-based substances that enter the gaseous phase by vaporization at 20 °C and 0.01 kPa [30]. Most VOCs are typically lipophilic liquids with high vapor pressure [31]. The emission of volatile organic compounds plays essential ecological and physiological roles for many organisms, such as fungi which release a broad spectrum of VOCs [32,33]. Fungi produce volatile compounds through their metabolic pathways [34,35] and they appear as intermediate and final products from both primary and secondary metabolism [36]. Fungal VOCs belong to different chemical groups such as monoterpenoids, sesquiterpenes, alcohols, aldehydes, aromatic compounds, esters, furans, hydrocarbons, ketones and others [31,33,37]. Since VOCs can spread through the atmosphere and the soil, they are ideal semiochemicals [38]. The aggregation pheromone sordidine, isolated from BW males [39,40] is used in insect traps as it is attractant for both sexes. BW adults are attracted to (2R,5S)-theaspirane, the active component of senescent banana leaves [41].

VOCs mediate interactions between organisms such as fungal–insect interactions [42]. Fungal production of VOCs is highly dynamic. The VOC profile of a species or strain may vary according to substrate, culture age, nutrient type, temperature and other environmental parameters [43,44,45]. VOCs profiles of *B. bassiana* and *M. anisopliae* have been correlated with their pathogenic activity [46]. Some fungal VOCs may have insecticidal or repellent activities. *Muscodor* spp. produces nitrosoamide which kills insects [47]. Naphthalene produced by *Muscodor vitigenus* (Daisy, Strobel, Ezra and W.M. Hess, 2002) is an insect repellent [48]. Some insects of various orders avoid fungal species of the Hypocreales, detecting their VOCs and negatively responding to them [42,49,50,51]. In addition, fungal VOCs show neurotoxicity in *Drosophila melanogaster* (Meigen, 1830) [52].

In this work, we isolate naturally occurring entomopathogenic fungi from banana soils to study their capability as biocontrol agents of BW. We analyze their VOC production to find a new approach for BW management. We also study BW behavior with these VOCs, searching for a repellent to be applied in the field.

## 2. Materials and Methods

### 2.1. Fungal Isolation from Banana Plantations Soils using Insect Baits

Soil samples from banana fields in the Canary Islands (Spain) (Table S1) were sampled as in Asensio et al. [53]. For each site, 2 kg of soil was collected from three points, one meter apart from each banana rhizome and between them. Soil was collected from 0–20 cm depth and stored at 4 °C until used.

We used *Galleria mellonella* (Linnaeus, 1756; Bichosa and Cebos Ramiro, Spain) larvae as living baits for entomopathogenic fungi (EF) isolation. Soil samples were screened using a one-millimeter sieve and then dried at room temperature. Forty grams of dried soil from each sample were placed in a Petri dish. Three Petri dishes (replicates) were prepared per soil sample. Ten milliliters of sterile distilled water (SDW) were then added to each Petri dish. Eight living larvae (L3-L4) of *G. mellonella* were buried per plate. Plates were sealed with Parafilm and incubated at 25 °C for 15 days in the dark. Plates were shaken periodically to favor contact between larvae and soil. Insects recovered from soil were surface-sterilized with 4% sodium hypochlorite for one minute. Insects were then rinsed three times in SDW (5 min each), and finally dried on sterile filter paper and placed in moist chambers for a week at 25 °C in the dark (five larvae per plate). Larvae were then plated on corn meal agar (CMA) with 50 μg/mL penicillin, 50 μg/mL streptomycin, 50 μg/mL rose Bengal and 1 mg/mL Triton X-100 [54]. Fungi on the larvae were isolated on CMA.

### 2.2. Morphological Identification of EF Isolates

Fifteen-day-old colonies from fungal isolates were used to prepare micro-cultures [55]. Fungal isolates were inoculated in 1 × 1 cm fragments of axenic CMA at their ends on sterile slides. CMA fragments were laid on top with a coverslip and placed in moist chambers. In this way, isolates were determined up to genus level using general taxonomic references [27,56,57].

### 2.3. Molecular Identification of Fungal Strains

EF isolates were grown in potato dextrose broth (PDB) for 5 days at 22 °C (120 rpm), filtered and dried to obtain mycelia samples, then they were frozen (liquid nitrogen), lyophilized and stored at −20 °C until use. For DNA extraction, we used 30 mg of mycelium for the acetyl-trimethyl-ammonium bromide (CTAB) method of O’Donnell et al. [58]. DNA quality was assessed with a ND-1000 Nanodrop (Wilmington, USA).

Internal transcribed spacers (ITS) region [59,60] and the translation elongation factor 1-alpha (TEF1α gen) [61] of EF isolates were amplified and sequenced. Primers used were: ITS-1F (5′TCCGTAGGTGAACCTGCGG3′) and ITS-4 (5′TCCTCCGCTTATTGATATGC3′); EFI-728F (5′CATCGAGAAGTTCGAGAAGG3′) and TEF-1R (5′GCCATCCTTGGGAGATACCAGC3′) fungal specific primers (0.5 μM). PCR was performed with the kit TaqPolimerase 2X (VWR) using the following PCR program: 95 °C for two minutes for the initial denaturation and, 35 cycles of 60 s at 95 °C, 30 s at 60 °C and 45 s at 72 °C to facilitate polymerization, and finally a five minutes extension at 72 °C was included. PCR products were purified using GeneJET Gel Extraction Kit (Thermo Scientific, Fisher) and sent for sequencing. We obtained consensus sequences of the amplified regions of the fungal strains, and they were compared by homology with other sequences in the NCBI database. Match sequences with homology values higher than 95% were used to assign the associated species checked in the NCBI database. The results were crosschecked with identifications obtained microscopically.

### 2.4. Pathogenicity Assays

The virulence of EF isolated from banana soils was evaluated by pathogenicity bioassays. We also included in the bioassays *B. bassiana* 203 strain (from naturally infected *Rynchophorous ferrugineus* (Olivier, 1790) adults in SE Spain, Daimès, Elche; CBS 121097) [62]. We evaluated EF virulence by testing the survival of *G. mellonella* larvae inoculated with a given strain of EF under laboratory conditions. Fifteen living larvae of *G. mellonella* were inoculated following the method of Ricaño et al. [63]. In the CMA plates bioassays, five larvae (L3-L4) were placed in 9 cm Petri dishes with a 24-day colony of each EF isolate grown in CMA. Insects were kept on the plate for 5 min, shaking the plate regularly to favor contact with the fungus. For controls, plates with non-inoculated CMA medium were used. Pathogenicity assays were carried out in duplicate. Insects were then placed in 9-cm Petri dishes in a moist chamber to evaluate the mortality every 12 h up to 20 days. In addition to this, dipping bioassays were carried out too, by making a 10-mL solution of 10^7^ conidia/mL and dipping the insects in the solution for five seconds. In these tests, we used *G. mellonella* larvae and *C. sordidus* adults, fifteen individuals of each species. Mortality was scored daily for 20 days.

Mantel–Cox test (*p* < 0.05, log-rank method, survival percentage analysis) was performed in GraphPad Prism 6 (version 6.01, 2012) using insect survival data. The virulence of EF isolates was evaluated by analyzing the evolution of the survival rate of the insects inoculated with EF versus the control treatment. Survival percentage curves were generated, and significant differences were studied and indicated.

### 2.5. Gas Chromatography—Mass Spectrometry (GC/MS) Analysis

Our two more virulent EF strains (*B. bassiana* 1TS11 and *M. robertsii* 4TS04; Table 1) and *B. bassiana* 203 were used for experiments. The nematophagous fungus *P. chlamydosporia* 123 strain (from eggs of *Heterodera avenae* (Wollenweber, 1924) in SW Spain, Seville; ATCC No. MYA-4875; CECT No. 20929) [64] was also included. Fungi were grown in 250-mL flasks with 75 g of autoclaved rice (*Oryza sativa* cv Redondo (Linnaeus, 1753); commercial animal food) [62] for 10, 20, 30, 40, 50 and 60 days at room temperature. Uninoculated rice was used as control.

GC/MS of fungal cultures was carried out using Solid-Phase Micro Extraction (SPME), with a fused silica fiber (1 cm; ∅ 0.110 mm) [65]. Cultures were processed for VOCs identification at 10, 20, 30, 40, 50 and 60 days after inoculation (dai). For each strain, five grams were sampled and placed in a vial (HS, crimb, FB, 20 mL, clr, cert, 100 PK, Agilent Technologies) hermetically capped by a pressure plug with a plastic membrane. Samples were then placed individually in a 60 °C thermostatic bath. The absorption of VOCs occurred by exposing the fiber of the holder to the headspace of the vial for 15 min. The holder was inserted into the GC injector (Agilent 5973 Network Mass Spectrometer - Agilent model 6890N gas chromatograph; column: DB624 30 m, 0.25 mm ID 1.4 µm, J&W Scientific) for a 4 min desorption at 150 °C in a splitless mode. The chromatography program used had an initial temperature of 35 °C for 5 min and a 3 °C/min increasing curve until 150 °C, then at that temperature for 1 min. Afterwards, a 5 °C/min increasing curve until 250 °C was used to finish the analysis (total analysis time was 38 min). The ionization source for electronic impact was 70 eV at 230 °C. A simple quadrupole was used as a detector at 150 °C. Wiley275 library was used for identifying VOCs.

After each chromatography run, the software generated a chromatogram and a list of VOCs. Data obtained were processed in order to obtain, for every strain, a list of the Total-VOCs characteristic of each fungus (T-VOCs) which a match ≥50% with the database entries. A second set included Major-VOCs (M-VOCs), which a match ≥50% and a peak-height (relative abundance) >100.000 ppm. Finally, the third set, which contained minor-VOCs (m-VOCs) for the compounds that have a match ≥50% and a peak-height between 100.000 and 20.000 ppm.

### 2.6. Olfactometer Bioassays

BW adults were collected from Tenerife (Canary Islands, Spain) using pheromone traps and kept in plastic boxes (40 × 30 × 21 cm) at 28 ± 0.5 °C in the dark. The plastic boxes contained a small container with distilled water, to maintain a moist environment, with a photoperiodic condition of 0:24 (L:D). Healthy BW adults collected from the field were used for bioassays. Fresh banana corm/pseudocorm (*Musa* sp.) pieces (ca. 16g/each) were used daily for olfactometer bioassays.

A two-way olfactometer (Figure S1) was used to evaluate the behavior of BW subjected to olfactory stimuli. At the end of the arms, two “odor chambers” [66] are placed, consisting of two glass tubes into which insert the olfactory stimuli to test. Tests were conducted by placing a single BW adult at the center of the straight arm. All insects tested had 10 min to move and make a choice or stay in the same place. After each test, the BW was stored for approximately one month, after that, individuals were changed for new ones. The olfactometer was rinsed after each test with ethanol, n-hexane and distilled water and dried with paper towels. Every test consisted of six replicates of 20 individuals each, which were reused every other time, from two boxes of 60 individuals each. The experiments were conducted with no airflow inside the olfactometer.

### 2.7. Environmental Conditions

In these tests, two conditions were analyzed, in which the attractant activity of the corm/pseudocorm was tested. We placed the natural attractant in one arm of the olfactometer, while nothing was placed in the other arm. We wanted to evaluate the effect of light (L) or darkness (D) on BW behavior and movement as BW mainly displays nocturnal habits [13]. Darkness tests were conducted in an off laminar flow hood covered with three layers of aluminum foil to ensure total darkness during the tests. Furthermore, starvation (S) was also tested. One population included BW fed ad libitum (No S), the other had BWs starved for at least a week (S). We evaluate the conditions in combination as follows: L-S, L-No S, D-S and D-No S. In this way, we can deduce in which conditions *C. sordidus* has the highest rate of movement for further bioassays.

### 2.8. Fungal VOCs Repellency

In this group, nine bioassays were conducted with starved BW in the dark (D-S). Seven fungal VOCs (C1-C7) (Table S2) and two BW repellents used in commercial banana plantations [67] (garlic, G. and colloidal sulphur, S.) were analyzed. Styrene (C1) and benzothiazole (C2) were selected because they repel *R. ferrugineus* [68]. *B. bassiana* 203 and 1TS11 both produced C1 and C2. Camphor (C3) and borneol (C4) were chosen for their repellence of insects [69,70,71]. C4 is produced by the *B. bassiana* strains, and C3 is produced only by *B. bassiana* 1TS11. We selected 1,3-dimethoxy-benzene (C5) and 1-octen-3-ol (C6) from *M. robertsii* and *P. chlamydosporia* because of their high abundance (M-VOCs). Finally, 3-cyclohepten-1-one was present in all fungal strains studied and in high abundance. 3-Cyclohepten-1-one was not commercially available and therefore bioassays were performed using the commercial isomer 2-cyclohepten-1-one (C7). Samples of all pure compounds were obtained from Sigma-Aldrich (St. Louis, MO, USA).

Of the VOCs identified using the fungal chromatograms, we tested the six pure substances commercially available for repellence against *C. sordidus*, except for 3-cyclohepten-1-one, because it was not commercially available. To replace 3-cyclohepten-1-one, we used 2-cyclohepten-1-one, the closest commercially available isomer. The substances were tested as pure compounds one by one and not in mixtures with each other. For the tests, in one arm of the olfactometer a piece of fresh corm/pseudocorm was placed, on the other arm 0.5 mL (C1, C2, C5, C6 and C7) or 0.5 g (C3 and C4) of the pure compounds were inoculated by placing them in a miracloth (Merck KGaA, Darmstadt, Germany) envelop (3.5 × 2.5 cm) with 2 g of silica gel (60A; 70–200 μ, Carlo Erba, Milan, Italy). The volume or weight of the compound was added directly to the silica gel. For the fresh garlic slices (local supermarket) and the colloidal sulphur (Sipcam Jardin S.L., Sipcam, Milan, Italy) 15.6 g of each substance was used.

### 2.9. Pheromone and Fungal VOCs Attractiveness

In these tests, four bioassays were carried out under D-S conditions to evaluate a sordidine-based pheromone (ECOSordidine30; ECOBERTURA, La Laguna, Tenerife, Spain) used in field traps. This aggregation pheromone, isolated from BW males [39,40] is an attractant for both sexes. The pheromone was compared in the olfactometer individually with no stimulus and C1 and C2, as described above. The pheromone concentration used was 1/16 (1.7 × 10^−4^ mL/cm^2^) of the concentration of the commercial formulation (0.27 mL/cm^2^). Commercial pheromone caused BW overstimulation and prevented movement.

Other bioassays were carried out in the same conditions as the previous ones. In this case, in one arm of the olfactometer, corm/pseudocorm was placed, whereas, in the other arm it was the pheromone alone or with C1 and C2.

### 2.10. Olfactometer Bioassays Data Treatment for Analysis

BWs could go to the arm containing corm/pseudocorm or pheromone—E1 (Figure S1). Others could go to the arm containing fungal VOCs, pheromone or no-stimulus—E2. Lastly, they could not move—EC. This triple ethological response has been evaluated with empirical mobility indexes, to summarize BWs behavior in the tests and to use them in the chi-square statistical tests.

We assume that a BW population placed in the olfactometer in the absence of stimuli would be uniformly distributed in the three possible choices maintaining a 1:1:1 (n_E1_ = n_E2_ = n_Ec_) ratio relationship. We also consider that the individuals remaining in the EC either, not respond to the stimulus or, are repelled by the substance tested. Therefore, three mobility rates can be formulated as follows: I_E1_ = n_E1_/N, I_E2_ = n_E2_/N and I_EC_ = n_EC_/N. Where, n_E1_ = number of individuals choosing E1, n_E2_ = number of individuals choosing E2, n_EC_ = number of individuals remaining in EC and N = total number of individuals tested. Finally, to obtain an index that takes into consideration all these relationships between groups of individuals, we have summarized theses rates into one, Index of Movement (IM): IM = (I_E1_ + I_E2_)/I_EC_ = (n_E1_ + n_E2_)/n_EC_. Where, 0 < IM < +∞, with n_EC_ ≠ 0. The closer to 0 the Index of Movement value is, the higher the motionless portion of the population is. Each of the six replicates (N = 20) originate an IM. ANOVA tests were conducted on all olfactometer bioassays using index data on the statistical software Rstudio.

## 3. Results

### 3.1. Entomopathogenic Fungi are Present in Soil from Banana Plantations

Soil sampling was performed in the Canary Islands where banana is the major crop. The number of samples collected per island was proportional to the area devoted to banana cultivation. In Tenerife, the largest island, with banana as a major crop, the most soil samples (23 out of 48) were taken. From 48 soil samples, we have detected the presence of entomopathogenic fungi (EF) in six soil samples (12.5%). All EF strains were isolated from soils of Tenerife (Table 1). All field locations with EF are under Integrated Production certification and drip irrigated (Table S1). We have isolated five fungal species from banana soils: *Beauveria bassiana* (1TS11) and *B. pseudobassiana* (19TS04), *Metarhizium robertsii* (4TS04) and *M. brunneum* (8TS21) and three *Simplicillium lamellicola* strains (2TS05, 5TS08 and 6TS01). We have identified them morphologically and by ITS and TEF1α sequencing. NCBI accession numbers are in Table 1. These isolates are deposited into the Spanish Type Culture Collection (CECT). Phylogenetic analysis confirms our species prediction (Figure S2).

### 3.2. Beauveria spp. from Banana Soils Are More Virulent than Metarhizium spp. to C. Sordidus

**Figure 1:**
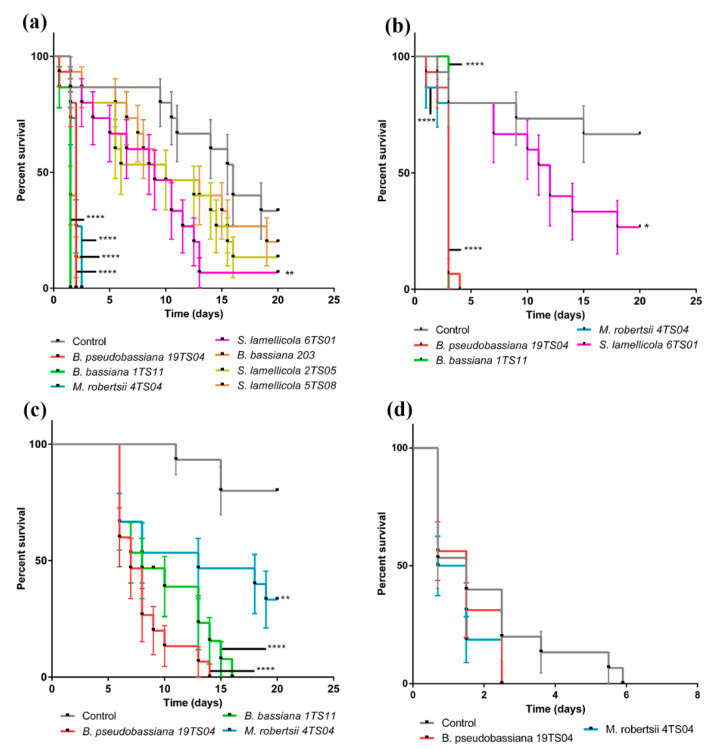
Entomopathogenic fungi isolated from banana crop soils are pathogenic over *G. mellonella* larvae and *C. sordidus* (*n* = 15). (**a**) *Beauveria* spp. and *M. robertsii* isolates are the most virulent on the larvae in the CMA plate bioassay. (**b**) In the dipping bioassay at 10^7^ conidia/mL the most virulent fungi are *Beauveria* spp. and *M. robertsii*. (**c**) *Beauveria* spp. isolated from banana crop soils of Tenerife are more virulent than *M. robertsii* 4TS04 on *C. sordidus* adults. (**d**) Entomopathogenic fungi isolated from banana crop soils are pathogenic over *C. sordidus* larvae. Asterisks indicate significant differences (* *p* < 0.05, ** *p* < 0.01, *** *p* < 0.001 and **** *p* < 0.0001) respect to the control.

The pathogenicity of EF isolates increased with humidity. *G. mellonella* larvae died four days faster under higher humidity conditions, studied by keeping the larvae under moist chamber conditions versus no extra humidity conditions (Figure 1A and Figure S3). We established these conditions to evaluate EF pathogenicity in further experiments.

*B. bassiana* 1TS11 and *B. pseudobassiana* 19TS04 were the most virulent strains, followed by *B. bassiana* 203 and *M. robertsii* 4TS04. These fungi induced *G. mellonella* full mortality after two days (Figure 1A). The mortality of larvae inoculated with *S. lamellicola* 2TS05 or 5TS08 did not differ significantly from controls. Therefore, these isolates were not included in further experiments.

In the *G. mellonella* dipping test, the most virulent strain was *M. robertsii* 4TS04, followed by *B. bassiana* 1TS11 and *B. pseudobassiana* 19TS04, with full mortality in less than five days (Figure 1B). Therefore, we selected these strains to analyze their VOCs.

In the *C. sordidus* pathogenicity test, the most virulent strain was *B. pseudobassiana* 19TS04, followed by *B. bassiana* 1TS11. BW adults were more resistant to EF than *G. mellonella* larvae. This is perhaps because BW cuticle is harder than that of *G. mellonella* larvae (Figure 1C). We also tested BW larvae sensitivity to EF. BW larvae exposed to *B. pseudobassiana* 19TS04 and *M. robertsii* 4TS04 fungal colonies died three days before the uninoculated larvae (Figure 1D). We noticed that BW larvae were very sensitive to manipulation and difficult to rear under laboratory conditions, so we made bioassays with a selection of EF only.

### 3.3. VOCs Production by Fungal Pathogens of Invertebrates

**Figure 2:**
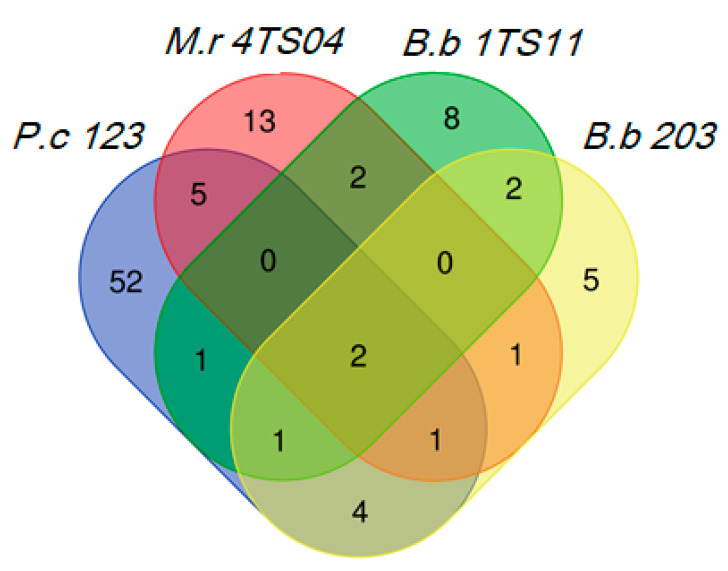
*P. chlamydosporia* 123 is the fungus that produces the most VOCs, followed by *M. robertsii* 4TS04. Only two compounds are produced by all the fungi analyzed by SPME-GC/MS. Abbreviations: *M.r 4TS04* = *M. robertsii* 4TS04, *B.b 1TS11* = *B. bassiana* 1TS11, *B.b 203* = *B. bassiana* 203 and *P.c 123* = *P. chlamydosporia* 123.

To evaluate VOCs, we selected the most pathogenic strains (*B. bassiana* 1TS11 and *M. robertsii* 4TS04) against *G. mellonella* larvae and BW adults. We also studied *B. bassiana* 203 isolated from red palm weevil [62] and the nematopathogenous fungus *P. chlamydosporia* 123 [64]. We found 97 volatile organic compounds produced by these fungal strains, during 60 days of growth (Figure 2, Table S3). Only 3-cyclohepten-1-one (C7) and 2-(2-ethoxyethoxy)-ethanol were produced by all fungal strains. *P. chlamydosporia* 123 was the largest VOCs producer with 52 unique compounds, followed by *M. robertsii* 4TS04 (13), *B. bassiana* 1TS11 (8) and *B. bassiana* 203 (5).

Regarding the most commonly produced VOCs, both *B. bassiana* strains (203 and 1TS11) emitted borneol (C4) and 1-octene. In the case of *M. robertsii* 4TS04 and *B. bassiana* 1TS11, they both produced 1,3-octadiene and (+-)-gymnomitrene. Regarding *M. robertsii* 4TS04 and *P. chlamydosporia* 123, they displayed the highest number of VOCs in common, such as 1-octen-3-ol (C6), 1-methyl-4-(1-methylethyl)-benzene and 1,3-dimethoxy-benzene (C5). *B. bassiana* 1TS11 and *P. chlamydosporia* 123, had only one VOC in common, 1-methylallyl(cyclooctatetraene)titanium. *M. robertsii* 4TS04 and *B. bassiana* 203, had only 4-fluoro-1,2-xylene in common. Finally, both *B. bassiana* 203 and *P. chlamydosporia* 123 emitted 2,4-octadiene, β-pinene, α-pinene and 3-octanone.

*B. bassiana* 203 generated 16 compounds characteristic of its metabolic profile (Figure 2). Of these, two were M-VOCs and the other five were m-VOCs (Table 2). 3-cyclohepten-1-one (C7) and borneol (C4) were present in all samplings carried out during the 60 days of growth of the fungus. Most of the compounds detected were found 20 days after inoculation (dai).

*B. bassiana* 1TS11 also generated 16 VOCs (Figure 2). Two belonged to the M-VOCs category, whereas nine were m-VOCs (Table 3). 3-cyclohepten-1-one (C7) was present in five of the samplings. Borneol (C4) was detected 10, 20, 40 and 50 dai. Benzothiazole (C2) was a m-VOC detected 20 dai only. Most compounds detected were found in the first sampling.

*M.robertsii* 4TS04 showed 24 VOCs in its metabolic profile (Figure 2). Of these, four were M-VOCs and eight were m-VOCs (Table 4). 3-cyclohepten-1-one (C7) was found from 10 to 30 dai, whereas 1-octen-3-ol (C6) was detected from 10 to 40 dai. 1,3-dimethoxy-benzene (C5) was only found at the first sampling. Most compounds were detected at 50 dai.

*P. chlamydosporia* 123 showed 66 VOCs (Figure 2). Of these, 12 compounds were M-VOCs and 42 compounds belonged to the m-VOCs (Table 5). 1,3-dimethoxy-benzene (C5) was found in the six samplings carried out during the growth of the fungus, while 1-octen-3-ol (C6) was detected 10, 20, 30 and 60 dai. 3-octanone was present 20, 40 and 50 dai, and 3-cyclohepten-1-one (C7) was found only at the first two samplings. Most compounds identified were detected at 40 and 50 dai.

### 3.4. Olfactometer Bioassays

#### 3.4.1. Effects of Environmental Conditions on BW Mobility

**Figure 3:**
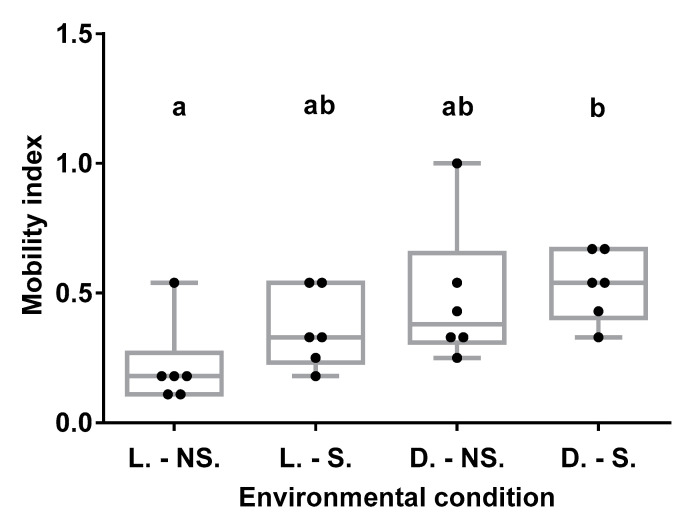
Darkness and starvation are the conditions that generate the most movement on *C. sordidus* (BW), they are the best environmental conditions for analyzing the mobility of BW in olfactometer assays. Abbreviations: L-NS = Light and no starvation, L-S = Light and starvation, D-NS = Darkness and no starvation and D-S = Darkness and starvation. Different letters indicate significant differences between treatments (*p* < 0.05).

Environmental conditions interfered with BW mobility (Figure 3) (χ^2^ = 17.952; df = 6; *p*-value = 0.006352; ANOVA: F-value = 3.305; *p*-value = 0.0413). The nocturnal activity of *C. sordidus* is evident from the higher mobility shown by individuals tested in the darkness, D-S and D-No S (IM_D-S_ = 0.53 and IM_D-No S_ = 0.48) compared to those conducted in the presence of light, L-S and L-No S (IM_L-S_ = 0.36 and IM_L-No S_ = 0.22). Individuals exposed to darkness-starvation conditions (D-S) showed the greatest mobility, reflected in the highest Index of Mobility (IM) (IM_D-S_ = 0.53), with significant differences (*p* < 0.05) with respect to light-no starvation conditions (L-No S) with the lowest IM (IM_L-No S_ = 0.22). Therefore, BW olfactometer bioassays (next section) were performed under darkness and starvation.

#### 3.4.2. Fungal VOCs Repel BW

**Figure 4:**
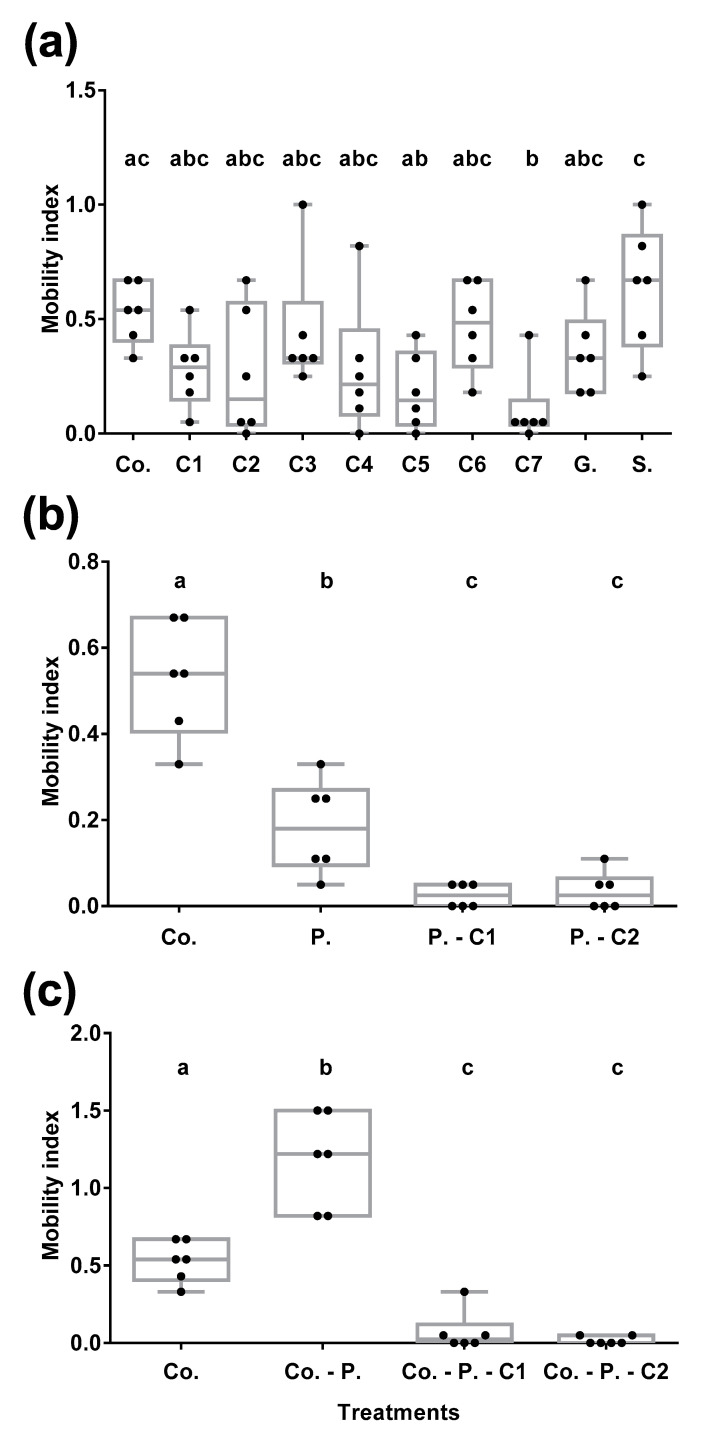
Evaluation of the mobility of *C. sordidus* (BW) subjected to different olfactory stimuli. (**a**) Effect of fungal VOCs and other compounds (technical repellents) versus no stimuli. C7 and C5 are the ones that reduced the most BW mobility. Abbreviations: Co. = Corm, C1 = styrene, C2 = benzothiazole, C3 = camphor, C4 = borneol, C5 = 1,3-dimethoxy-benzene, C6 = 1-octen-3-ol, C7 = 2-cyclohepten-1-one, G = garlic, S = colloidal sulphur. (**b**) Effect of fungal VOCs on *C. sordidus* pheromone attractiveness. C1 and C2 mask pheromone attractiveness on BW. Co. = Corm vs. no stimuli; P. = Pheromone vs. no stimuli; P.-C1 = Pheromone vs. C1; P.-C2 = Pheromone vs. C2 (**c**) Effect of banana corm, BW aggregation pheromone and fungal VOCs attractiveness to *C. sordidus*. C1 and C2 mask banana corm and pheromone attractiveness on BW. Co. = Corm vs. no stimuli; Co.-P. = Corm vs. Pheromone; Co.-P.-C1 = Corm vs. Pheromone + C1; Co.-P.-C2 = Corm vs. Pheromone + C2. Different letters indicate significant differences between treatments (*p* < 0.05).

*C. sordidus* mobility was influenced by fungal VOCs (Table S2) and other compounds (technical repellents) tested (Figure 4A) (χ^2^ = 60.881; df = 18; *p*-value = 1.473 × 10^−6^; ANOVA: F-value = 3.388; *p*-value = 0.0026). All fungal VOCs and technical repellents, except for colloidal sulphur (S.), reduced BW movement compared to the control (corm only, no repellents). However, significant differences (*p* < 0.05) were only observed with the commercially available compound 2-cyclohepten-1-one (C7) we used because it is an isomer of 3-cyclohepten-1-one. Both isomers (3-cyclohepten-1-one and 2-cyclohepten-1-one) may impact BW differently. Sulphur showed the highest IM (IM_S_ = 0.64) compared to the control (IM_D-S_ = 0.53), being significantly different (*p* < 0.05) with 1,3-dimethoxy-benzene (C5) and C7. The compound that mostly reduced BW mobility was C7 (IM_C7_ = 0.11), followed by C5 (IM_C5_ = 0.18) produced by *P. chlamydosporia* 123 and *M. roberstii* 4TS04 only. Benzothiazole (C2) (IM_C2_ = 0.26) and styrene (C1) (IM_C1_ = 0.28) were the following ones, showing a decrease in BW mobility.

#### 3.4.3. Fungal VOCs Mask Pheromone and Banana Corm BW Attractiveness

*C. sordidus* behavior was influenced by sordidine (BW aggregation pheromone, P), styrene and benzothiazole (C1 and C2, respectively fungal VOCs) (Figure 4B) (χ^2^ = 23.221; df = 4; *p*-value = 1.14 × 10^−4^). C1 and C2 significantly modified *C. sordidus* mobility with respect to P (ANOVA: F-value = 9.769; *p*-value = 0.0019). VOCs presence generated an IM (IM_PC1_ = 0.03 and IM_PC2_ = 0.04) lower than that of the pheromone (IM_P_ = 0.18). C1 and C2 therefore repelled BW.

Fungal VOCs C1 and C2, combined with pheromone (IM_C-PC1_ = 0.07 and IM_C-PC2_ = 0.02) masked pheromone BW attractiveness. This was reflected by the lower IM of BW of VOCs-pheromone stimuli compared to pheromone alone (IM_C-P_ = 1.18) versus corm (Figure 4C) (χ^2^ = 123.43; df = 4; *p*-value = 2.2 × 10^−16^; ANOVA: F-value = 54.13; *p*-value = <0.0001).

## 4. Discussion

Bananas are essential for food security in tropical and subtropical countries, being one of the best-known, consumed and cultivated fruits [72,73]. *Cosmopolites sordidus* is a major pest of bananas. It causes more crop destruction than any other arthropod pest in all banana producing countries [5]. BW-resistant banana plants do not cover main commercial cultivars, making this pest a severe problem [74,75,76].

Biological control agents such as entomopathogenic fungi could be used for BW management [5,77]. However, *C. sordidus* larvae and adults spend most of their life cycle within the banana plant, where it is hard to target them using EF conidia. Moreover, the adults move from plant to plant, hiding in the leaf litter, complicating the use of EF conidia. Lopes et al. [78] reported that *C. sordidus* adults are less sensitive to *B. bassiana* in auto-infection systems (mortality ranged between 21.7 and 1%) than other beetles, like *Cylas formicarius* (Fabricius, 1798) and *Ips typographus* (Linnaeus, 1758).

In this work, we take an alternative approach for BW biomanagement using fungal VOCs. BWs have efficient search mechanisms based on their antennae, specialized primary chemo and mechanoreceptors, which are crucial to ensure the survival and reproduction of BWs in the environment [13].

Fungi produce volatile compounds [34,35]. Some of them act as attractants and/or repellents for insects and other invertebrates [42,46,47,48,49,50,51,52]. These compounds may alert the insect about possible partners, food, suitable places to lay their eggs or dangers that should be avoided. Therefore, any chemical that could interrupt and modify the behaviour of the BW and, in general, its searching ability for the host (*Musa* sp.) could serve as a tool for BW sustainable management.

We have isolated entomopathogenic fungi from banana crop soils in Tenerife (Canary Islands, Spain), where BW infestations are documented, looking for VOCs that are repellent to BW. *Beauveria bassiana* (1TS11) and *M. robertsii* (4TS04), both pathogenic to BW, were selected for VOC analysis from this survey. These fungi are common in agricultural fields [79] and banana crops [78]. Since *B. bassiana* 203 produces VOCs repellent to *R. ferrugineus* [68], it was included in this study. *Pochonia chlamydosporia* 123, a nematophagous fungus closely related to *M. anisopliae* [20,21], with a large array of secondary metabolites [80] was also tested for VOC production. Genomic studies support that some *Metarhizium* species and *P. chlamydosporia* have a single ancestral joint [21].

These fungi produce a total of 97 VOCs. The VOC 3-cyclohepten-1-one, one of the main VOCs produced by all fungal strains [81], was not tested on *C. sordidus* because it was not commercially available. However, its isomer 2-cyclohepten-1-one (C7) reduced BW mobility most among the seven VOCs tested. Previous work found that stereoisomers and isomers can be equally repellent to insects, while other studies describe that insect repellence was different for various piperidine isomers. We do not know how 3-cyclohepten-1-one acts as a repellent, but the most similar isomer, 2-cyclohepten-1-one, repels BW. We decided to test 2-cyclohepten-1-one as a first approach to validate cycloheptenes as BW repellents. The synthesis of 3-cyclohepten-1-one in the laboratory is complex, expensive, and tedious (involving oxidation and ring closure). 3-cyclohepten-1-one is also the most unstable cycloheptene isomer because it isomerizes the double bond to form the more stable 2-cyclohepten-1-one (used in this study as C7). Therefore, 3-cyclohepten-1-one is unavailable in the quantities required for bioassays, let alone field trials. Electroantennography of experimentally synthesized 3-cyclohepten-1-one could, however, be performed in future studies.

The 1,3-dimethoxy-benzene (C5) from *P. chlamydosporia* and *M. robertsii* cultures was the second most repellent VOC to *C. sordidus*. Camphor (C3) and borneol (C4), produced by *B. bassiana* and *B. pseudobassiana*, moderately reduce BW movement. These are known insect repellents [69,70,71,82]. 1-octen-3-ol (C6), responsible for the odour of many fungi [83,84], is a milder repellent to *C. sordidus*. Styrene (C1) and benzothiazole (C2) are also repellent to *C. sordidus*. They can reduce banana corm and pheromone (sordidine) attractiveness to BW.

## 5. Conclusions

In this work, we have identified new VOCs from EF and a nematophagous fungus that can be used as BW repellents. 1,3-Dimethoxy-benzene (C5), styrene (C1), benzothiazole (C2), camphor (C3), borneol (C4), 1-octen-3-ol (C6) and 2-cyclohepten-1-one (C7) reduced BW mobility and can therefore be considered for BW management. Because 3-cyclohepten-1-one is unstable, difficult to synthesise, and not commercially available, we evaluated the repellence of 2-cyclohepten-1-one. In addition, C1 and C2 mask sordidine, the commercial BW aggregation pheromone. Tests should be conducted to determine the effect of selected VOCs as BW repellents in the field. Using VOCs and slow-release polymer matrices would improve VOCs’ performance and durability in the field. Implementing the technologies associated with the dispersion of these repellents could produce advancements in the agrobiotechnological sustainability of world banana cultivation. The economic importance of BW at a global level justifies the continuation of research in identifying new molecules and technologies for the genesis of these new means of bio-management. Managing *C. sordidus* in an integrated way could contribute to the increase in banana production, significantly contributing to the increase in global food production, given the extent and importance of this crop.

## 6. Patents

The results regarding fungal VOCs have been patented in the Spanish Office of Brands and Patents (OEPM) with patent number P201930831, with Luis Vicente Lopez-Llorca, Ana Lozano-Soria, Ugo Picciotti, Federico Lopez-Moya and Javier Lopez-Cepero as inventors.

## Figures and Tables

**Table 1:** Entomopathogenic fungi isolated from Tenerife Island banana crops soils.

Fungi spp.	Code	CECT Number	NCBI Number	Soil Code	Fungal Genera	Blastn	
						ITS	Tef1a
*Simplicillium lamellicola*	*A.l.6TS01*	CECT 21125	MK156720.1	TS01	*Simplicillium sp.*	*Simplicillium lamellicola*	*-*
*Metarizhium robertsii*	*M.r.4TS04*	CECT 21126	MK156715.1	TS04	*Metarhizium sp.*	*Metarhizium robertsii*	*M. robertsii*
*Beauveria pseudobassiana*	*B.p.19TS04*	CECT 21122	MK156716.1	TS04	*Beauveria sp.*	*Beauveria pseudobassiana*	*B. pseudobasiana*
*Simplicillium lamellicola*	*A.l.2TS05*	CECT 21123	MK156718.1	TS05	*Simplicillium sp.*	*Simplicillium lamellicola*	*-*
*Simplicillium lamellicola*	*A.l.5TS08*	CECT 21124	MK156719.1	TS08	*Simplicillium sp.*	*Simplicillium lamellicola*	*-*
*Beauveria bassiana*	*B.b.1TS11*	CECT 21121	MK156717.1	TS11	*Beauveria sp.*	*Beauveria bassiana*	*B. bassiana*
*Metarhizium brunneum*	*M.b.8TS21*	-	MT703853	TS21	*Metarhizium sp.*	*Metarhizium brunneum*	*M. brunneum*

**Table 2:** M-VOCs and m-VOCs produced by *B. bassiana* 203 during 60 days of growth. Abbreviations: VOC = volatile organic compound, M-VOCs = major VOCs, m-VOCs = minor VOCs, d = days after inoculation.

*Beauveria bassiana 203*	10d	20d	30d	40d	50d	60d
**M-VOCs**						
3-cyclohepten-1-one	X	X	X	X	X	X
4-fluoro-1,2-xylene						X
**m-VOCs**						
p-Trimethylsilyloxyphenyl-bis(trimethylsilyloxy)ethane		X				
Borneol	X	X	X	X	X	X
3-octanone		X				
(2R*,6R*,8AS*)-6-hydroxyedulan		X				
β-pinene			X			

**Table 3:** M-VOCs and m-VOCs produced by *B. bassiana* 1TS11 during 60 days of growth. Abbreviations: VOC = volatile organic compound, M-VOCs = major VOCs, m-VOCs = minor VOCs, d = days after inoculation.

*Beauveria bassiana 1TS11*	10d	20d	30d	40d	50d	60d
**M-VOCs**						
1,3-octadiene					X	
3-cyclohepten-1-one	X (m-VOCs)	X	X	X		X
**m-VOCs**						
Methyl-1,4-dioxide-pyrazine	X					
2-methyl-2-bornene	X					
2-(2-ethoxyethoxy)-ethanol			X			
2,2,4,4,6,8,8-heptamethyl-nonane	X					
1-methyl-1H-1,2,4-triazole	X					
Borneol	X	X		X	X	
Benzothiazole			X			
5-ethyl-3-hydroxy-4-methyl-2(5H)-furanone			X			
(+-)-gymnomitrene						X

**Table 4:** M-VOCs and m-VOCs produced by *M. robertsii* 4TS04 during 60 days of growth. Abbreviations: VOC = volatile organic compound, M-VOCs = major VOCs, m-VOCs = minor VOCs, d = days after inoculation.

*Metarizhium robertsii 4TS04*	10d	20d	30d	40d	50d	60d
**M-VOCs**						
3-cyclohepten-1-one	X	X	X			
1-octen-3-ol	X(m-VOCs)	X	X	X(m-VOCs)		
4-fluoro-1,2-xylene		X				X
1,3-dimethoxy-benzene	X					
**m-VOCs**						
1,3-octadiene				X		
3,5-dimethyl-2-propylthiophene					X	
1-β-pinene						X
1-methyl-4-(1-methylethyl)-benzene			X			
2-amino-3,5-dibromo-6-methylpyridine					X	
4-octen-3-ol					X	
2,4-bis(1,1-dimethylethyl)-phenol		X				
5-oxoisoboldine					X	

**Table 5:** M-VOCs and m-VOCs produced by *P. clamydosphoria* 123 during 60 days of growth. Abbreviations: VOC = volatile organic compound, M-VOCs = major VOCs, m-VOCs = minor VOCs, d = days after inoculation.

*Pochonia clamydosphoria 123*	10d	20d	30d	40d	50d	60d
**M-VOCs**						
Dimethyldisulfide				X	X	
3-methyl-2-pentanone				X		
3-cyclohepten-1-one	X	X				
Propyl-cyclohexane						X
Dimethyl-trisulfide					X	
1-octen-3-ol	X	X	X			X
3-octanone		X		X	X	
2-dodecanone				X	X	
1-methylallyl(cyclooctatetraene)titanium					X	
1-phenyl-2-propanone				X	X	
1,3-dimethoxy-benzene	X	X	X	X	X	X
β-elemene						X
**m-VOCs**						
2-butanone				X	X	
3-methylbutanal			X		X	X
3-hydroxy-2-butanone		X				
2-methylbutanal			X			
2,4-octadiene		X				
4-methyl-3-hexanone				X		
Nonane	X					
(1-methylethenyl) cyclopropene			X			
5-methyl-2-hexanone				X		
2-butoxy-ethanol				X		
Propionoin				X	X	
2,4-dimethyl-3-hexene				X		
3,4-dimethyl-2-hexene					X	
Trans-3,4-dimethyl-2-hexene			X			
β-pinene						X
Anisole	X					
2-isopropyl-5-oxohexanal					X	
6-methyl-2-heptanone				X		
Trimethyl-pyrazine					X	
2-methyl-2-bornene	X					
2,4-dimethyl-methyl ester-hexanoic acid		X				
1-methyl-4-(1-methylethyl)-benzene				X		
Isocyano-benzene					X	
2-(2-ethoxyethoxy)-ethanol			X	X		
2,4-dimethylfuran						X
2-dodecanone				X	X	
2-methyl-2-(2-methyl-2-butenyl)-furan				X		
Benzenemethanol					X	
3-hydroxymandelic acid ethyl ester di-TMS		X			X	
4-hydroxymandelic acid ethyl ester di-TMS						X
4-trimethylsilyl-9,9-dimethyl-9-silafluorene	X					
6,7-dimethoxy-2,2-dimethyl-2H-1-benzopyran	X					
Bicyclo[2 .2.1]heptane-2-carboxylic acid	X					
2-amino-4-methylpyrimidine		X				
2,4-bis(1,1-dimethylethyl)-phenol	X					
2-undecanone					X	
2-octanone				X		
1-ethyl-2-methyl-cyclohexane				X		
16-oxosalutaridine					X	X
5-acetyl-2-hydrazino-4-methylpyridine				X		
Cyclotetradecane				X		
trans-β-farnesene		X				

## Supplementary Materials

The following are available online at https://www.mdpi.com/2075-4450/11/8/509/s1, Table S1: Soil collection from banana fields in the Canary Islands. Figure S1: Two-way olfactometer structure. BW behavior in the olfactometer. Table S2: Fungal volatile organic compounds selected for the olfactometry bioassays. Figure S2: Molecular Phylogenetic analysis of the ITS region by Maximum Likelihood method. Evolutionary analyses were conducted in MEGA X. Figure S3: *B. pseudobassiana* 19TS04, *B. bassiana* 1TS11, *M. robertsii* 4TS04, *B. bassiana* 203 and *S. lamellicola* 6TS01 are significantly virulent on *G. mellonela* larvae at room humidity conditions. Table S3. Volatile organic compounds produced by the fungi analyzed, in all the times measured.
