# Correction: Lozano-Soria et al. Volatile Organic Compounds from Entomopathogenic and Nematophagous Fungi, Repel Banana Black Weevil (*Cosmopolites sordidus*). *Insects* 2020, *11*, 509

**DOI:** 10.3390/insects15030175

**Published:** 2024-03-05

**Authors:** Ana Lozano-Soria, Ugo Picciotti, Federico Lopez-Moya, Javier Lopez-Cepero, Francesco Porcelli, Luis Vicente Lopez-Llorca

**Affiliations:** 1 Department of Marine Science and Applied Biology, Laboratory of Plant Pathology, University of Alicante, 03080 Alicante, Spain; federico.lopez@ua.es (F.L.-M.); lv.lopez@ua.es (L.V.L.-L.); 2 Department of Soil, Plant, and Food Sciences—(DiSSPA), University of Bari Aldo Moro, 70126 Bari, Italy; francesco.porcelli@uniba.it; 3 Dyrecta Lab Istituto di Ricerca, 70014 Conversano, Bari, Italy; 4 Technical Department of Coplaca, 38001 Tenerife, Spain; cepero@coplaca.org

## Figure Legend

In the original publication [1], there was a mistake in the legend for Figure 4. The correct name for compound C7 is 2-cyclohepten-1-one. The correct legend appears below. The authors state that the scientific conclusions are unaffected. This correction was approved by the Academic Editor. The original publication has also been updated.

Corrected legend: **Figure 4.** Evaluation of the mobility of *C. sordidus* (BW) subjected to different olfactory stimuli. (**a**) Effect of fungal VOCs and other compounds (technical repellents) versus no stimuli. C7 and C5 are the ones that reduced the most BW mobility. Abbreviations: Co. = Corm, C1 = styrene, C2 = benzothiazole, C3 = camphor, C4 = borneol, C5 = 1,3-dimethoxy-benzene, C6 = 1-octen-3-ol, C7 = 2-cyclohepten-1-one, G = garlic, S = colloidal sulphur. (**b**) Effect of fungal VOCs on *C. sordidus* pheromone attractiveness. C1 and C2 mask pheromone attractiveness on BW. Co. = Corm vs. no stimuli; P. = Pheromone vs. no stimuli; P.-C1 = Pheromone vs. C1; P.-C2 = Pheromone vs. C2 (**c**) Effect of banana corm, BW aggregation pheromone and fungal VOCs attractiveness to *C. sordidus*. C1 and C2 mask banana corm and pheromone attractiveness on BW. Co. = Corm vs. no stimuli; Co.-P. = Corm vs. Pheromone; Co.-P.-C1 = Corm vs. Pheromone + C1; Co.-P.-C2 = Corm vs. Pheromone + C2. Different letters indicate significant differences between treatments (*p* < 0.05).

## Text Correction

There was an error in the original publication. One of the VOCs found in the volatilome of entomopathogenic and nematophagous fungi was 3-cyclohepten-1-one (also called C7 in the article). The olfactometric tests with banana weevil (*Cosmopolites sordidus*) were conducted instead with 2-cyclohepten-1-one. The reason being that this was the only isomer commercially available rather than 3-cyclohepten-1-one.

A correction has been made to Abstract.

Corrected paragraph: **Abstract:** Fungal Volatile Organic Compounds (VOCs) repel banana black weevil (BW), *Cosmopolites sordidus* (Germar, 1824), the key-pest of banana [*Musa* sp. (Linnaeus, 1753)]. The entomopathogens *Beauveria bassiana* (Bb1TS11) and *Metarhizium robertsii* (Mr4TS04) were isolated from banana plantation soils using an insect bait. Bb1TS11 and Mr4TS04 were pathogenic to BW adults. Bb1TS11, Bb203 (from infected palm weevils), Mr4TS04 and the nematophagous fungus *Pochonia clamydosporia* (Pc123), were tested for VOCs production. VOCs were identified by Gas Chromatography/Mass Spectrometry–Solid-Phase Micro Extraction (GC/MS-SPME). GC/MS-SPME identified a total of 97 VOCs in all strains tested. Seven VOCs (styrene, benzothiazole, camphor, borneol, 1,3-dimethoxy-benzene, 1-octen-3-ol and 3-cyclohepten-1-one) were selected for their abundance or previous record as insect repellents. In olfactometry bioassays, BW-starved adults in the dark showed the highest mobility to banana corm. 2-cyclohepten-1-one (C7), commercially available isomer of 3-cyclohepten-1-one, is the best BW repellent (*p* < 0.05), followed by 1,3-dimethoxy-benzene (C5). The rest of the VOCs have a milder repellency to BW. Styrene (C1) and benzothiazole (C2) (known to repel palm weevil) block the attraction of banana corm and BW pheromone to BW adults in bioassays. Therefore, VOCs from biocontrol fungi can be used in future studies to biomanage BW in the field.

A correction has been made to 2. Materials and Methods, 2.8. Fungal VOCs Repellency.

Corrected paragraph: In this group, nine bioassays were conducted with starved BW in the dark (D-S). Seven fungal VOCs (C1-C7) (Table S2) and two BW repellents used in commercial banana plantations [67] (garlic, G. and colloidal sulphur, S.) were analyzed. Styrene (C1) and benzothiazole (C2) were selected because they repel *R. ferrugineus* [68]. *B. bassiana* 203 and 1TS11 both produced C1 and C2. Camphor (C3) and borneol (C4) were chosen for their repellence of insects [69–71]. C4 is produced by the B. bassiana strains, and C3 is produced only by *B. bassiana* 1TS11. We selected 1,3-dimethoxy-benzene (C5) and 1-octen-3-ol (C6) from *M. robertsii* and *P. chlamydosporia* because of their high abundance (M-VOCs). Finally, 3-cyclohepten-1-one was present in all fungal strains studied and in high abundance. 3-Cyclohepten-1-one was not commercially available and therefore bioassays were performed using the commercial isomer 2-cyclohepten-1-one (C7). Samples of all pure compounds were obtained from Sigma-Aldrich (St. Louis, MO, USA).

Of the VOCs identified using the fungal chromatograms, we tested the six pure substances commercially available for repellence against *C. sordidus*, except for 3-cyclohepten-1-one, because it was not commercially available. To replace 3-cyclohepten-1-one, we used 2-cyclohepten-1-one, the closest commercially available isomer. The substances were tested as pure compounds one by one and not in mixtures with each other. For the tests, in one arm of the olfactometer a piece of fresh corm/pseudocorm was placed, on the other arm 0.5 mL (C1, C2, C5, C6 and C7) or 0.5 g (C3 and C4) of the pure compounds were inoculated by placing them in a miracloth (Merck KGaA, Darmstadt, Germany) envelop (3.5 × 2.5 cm) with 2 g of silica gel (60A; 70–200 μ, Carlo Erba, Milan, Italy). The volume or weight of the compound was added directly to the silica gel. For the fresh garlic slices (local supermarket) and the colloidal sulphur (Sipcam Jardin S.L., Sipcam, Milan, Italy) 15.6 g of each substance was used.

A correction has been made to 3. Results, 3.4. Olfactometer Bioassays, 3.4.2. Fungal VOCs Repel BW.

Corrected paragraph: *C. sordidus* mobility was influenced by fungal VOCs (Table S2) and other compounds (technical repellents) tested (Figure 4A) (χ^2^ = 60.881; df = 18; *p*-value = 1.473 × 10^−6^; ANOVA: F-value = 3.388; *p*-value = 0.0026). All fungal VOCs and technical repellents, except for colloidal sulphur (S.), reduced BW movement compared to the control (corm only, no repellents). However, significant differences (*p* < 0.05) were only observed with the commercially available compound 2-cyclohepten-1-one (C7) we used because it is an isomer of 3-cyclohepten-1-one. Both isomers (3-cyclohepten-1-one and 2-cyclohepten-1-one) may impact BW differently. Sulphur showed the highest IM (IM_S_ = 0.64) compared to the control (IM_D-S_ = 0.53), being significantly different (*p* < 0.05) with 1,3-dimethoxy-benzene (C5) and C7. The compound that mostly reduced BW mobility was C7 (IM_C7_ = 0.11), followed by C5 (IM_C5_ = 0.18) produced by *P. chlamydosporia* 123 and *M*. *roberstii* 4TS04 only. Benzothiazole (C2) (IM_C2_ = 0.26) and styrene (C1) (IM_C1_ = 0.28) were the following ones, showing a decrease in BW mobility.

A correction has been made to Section 4. Discussion.

Corrected paragraph: Bananas are essential for food security in tropical and subtropical countries, being one of the best-known, consumed and cultivated fruits [72,73]. *Cosmopolites sordidus* is a major pest of bananas. It causes more crop destruction than any other arthropod pest in all banana producing countries [5]. BW-resistant banana plants do not cover main commercial cultivars, making this pest a severe problem [74–76].

Biological control agents such as entomopathogenic fungi could be used for BW management [5,77]. However, *C. sordidus* larvae and adults spend most of their life cycle within the banana plant, where it is hard to target them using EF conidia. Moreover, the adults move from plant to plant, hiding in the leaf litter, complicating the use of EF conidia. Lopes et al. [78] reported that *C. sordidus* adults are less sensitive to *B. bassiana* in auto-infection systems (mortality ranged between 21.7 and 1%) than other beetles, like *Cylas formicarius* (Fabricius, 1798) and *Ips typographus* (Linnaeus, 1758).

In this work, we take an alternative approach for BW biomanagement using fungal VOCs. BWs have efficient search mechanisms based on their antennae, specialized primary chemo and mechanoreceptors, which are crucial to ensure the survival and reproduction of BWs in the environment [13].

Fungi produce volatile compounds [34,35]. Some of them act as attractants and/or repellents for insects and other invertebrates [42,46–52]. These compounds may alert the insect about possible partners, food, suitable places to lay their eggs or dangers that should be avoided. Therefore, any chemical that could interrupt and modify the behaviour of the BW and, in general, its searching ability for the host (*Musa* sp.) could serve as a tool for BW sustainable management.

We have isolated entomopathogenic fungi from banana crop soils in Tenerife (Canary Islands, Spain), where BW infestations are documented, looking for VOCs that are repellent to BW. *Beauveria bassiana* (1TS11) and *M. robertsii* (4TS04), both pathogenic to BW, were selected for VOC analysis from this survey. These fungi are common in agricultural fields [79] and banana crops [78]. Since *B. bassiana* 203 produces VOCs repellent to *R. ferrugineus* [68], it was included in this study. *Pochonia chlamydosporia* 123, a nematophagous fungus closely related to *M. anisopliae* [20,21], with a large array of secondary metabolites [80] was also tested for VOC production. Genomic studies support that some *Metarhizium* species and *P. chlamydosporia* have a single ancestral joint [21].

These fungi produce a total of 97 VOCs. The VOC 3-cyclohepten-1-one, one of the main VOCs produced by all fungal strains [81], was not tested on *C. sordidus* because it was not commercially available. However, its isomer 2-cyclohepten-1-one (C7) reduced BW mobility most among the seven VOCs tested. Previous work found that stereoisomers and isomers can be equally repellent to insects, while other studies describe that insect repellence was different for various piperidine isomers. We do not know how 3-cyclohepten-1-one acts as a repellent, but the most similar isomer, 2-cyclohepten-1-one, repels BW. We decided to test 2-cyclohepten-1-one as a first approach to validate cycloheptenes as BW repellents. The synthesis of 3-cyclohepten-1-one in the laboratory is complex, expensive, and tedious (involving oxidation and ring closure). 3-cyclohepten-1-one is also the most unstable cycloheptene isomer because it isomerizes the double bond to form the more stable 2-cyclohepten-1-one (used in this study as C7). Therefore, 3-cyclohepten-1-one is unavailable in the quantities required for bioassays, let alone field trials. Electroantennography of experimentally synthesized 3-cyclohepten-1-one could, however, be performed in future studies.

The 1,3-dimethoxy-benzene (C5) from *P. chlamydosporia* and *M. robertsii* cultures was the second most repellent VOC to *C. sordidus*. Camphor (C3) and borneol (C4), produced by *B. bassiana* and *B. pseudobassiana*, moderately reduce BW movement. These are known insect repellents [69–71,82]. 1-octen-3-ol (C6), responsible for the odour of many fungi [83,84], is a milder repellent to *C. sordidus*. Styrene (C1) and benzothiazole (C2) are also repellent to *C. sordidus*. They can reduce banana corm and pheromone (sordidine) attractiveness to BW.

A correction has been made to Section 5. Conclusions.

In this work, we have identified new VOCs from EF and a nematophagous fungus that can be used as BW repellents. 1,3-Dimethoxy-benzene (C5), styrene (C1), benzothiazole (C2), camphor (C3), borneol (C4), 1-octen-3-ol (C6) and 2-cyclohepten-1-one (C7) reduced BW mobility and can therefore be considered for BW management. Because 3-cyclohepten-1-one is unstable, difficult to synthesise, and not commercially available, we evaluated the repellence of 2-cyclohepten-1-one. In addition, C1 and C2 mask sordidine, the commercial BW aggregation pheromone. Tests should be conducted to determine the effect of selected VOCs as BW repellents in the field. Using VOCs and slow-release polymer matrices would improve VOCs’ performance and durability in the field. Implementing the technologies associated with the dispersion of these repellents could produce advancements in the agrobiotechnological sustainability of world banana cultivation. The economic importance of BW at a global level justifies the continuation of research in identifying new molecules and technologies for the genesis of these new means of bio-management. Managing *C. sordidus* in an integrated way could contribute to the increase in banana production, significantly contributing to the increase in global food production, given the extent and importance of this crop.

The authors state that the scientific conclusions are unaffected. This correction was approved by the Academic Editor. The original publication has also been updated.
