# Supplementary material for: Volatile Organic Compounds from Entomopathogenic and Nematophagous Fungi, Repel Banana Black Weevil (Cosmopolites sordidus)
Source: Insects. 2020 Aug 6;11(8):509. doi: 10.3390/insects11080509 (PMC7469225; doi:10.3390/insects11080509)
Supplement: Supplementary file 1 [file insects-11-00509-s001.pdf]

Supplementary material

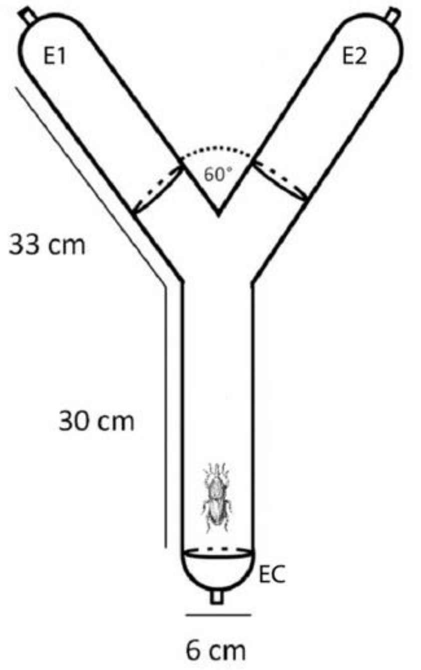

Figure S1. Two-way olfactometer structure. BW behaviour in the olfactometer: E1 represents individuals choosing one stimuli, E2 represents individuals choosing the other stimuli or absence of stimulus and EC indicate individuals not moving from the inicial place.

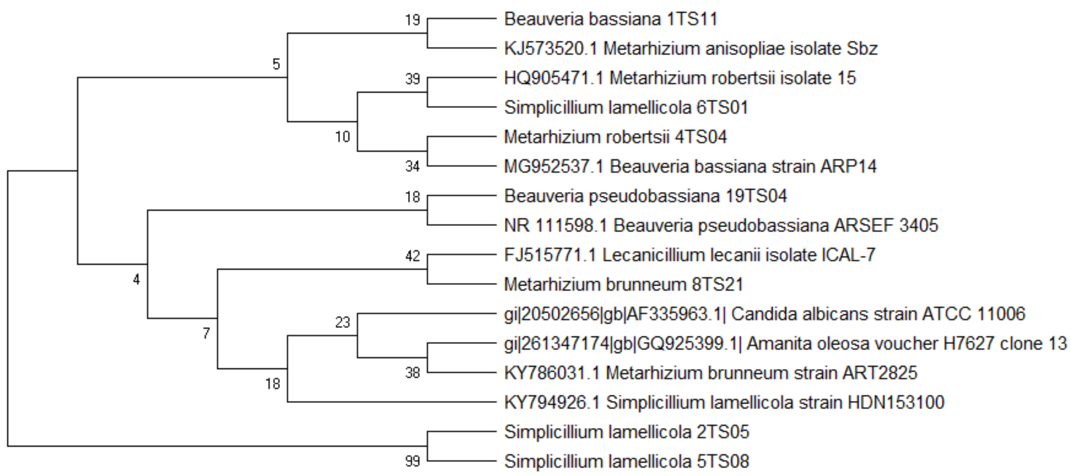

Figure S2. Molecular Phylogenetic analysis of the ITS region by Maximum Likelihood method (Boostrap consensus tree of 800 replicates), based on the Tamura-Nei model. Branches corresponding to partitions reproduced in less than 50% bootstrap replicates are collapsed. The percentage of replicate trees in which the associated taxa clustered together in the bootstrap test are shown next to the branches [1]. Evolutionary analyses were conducted in MEGA X [2].

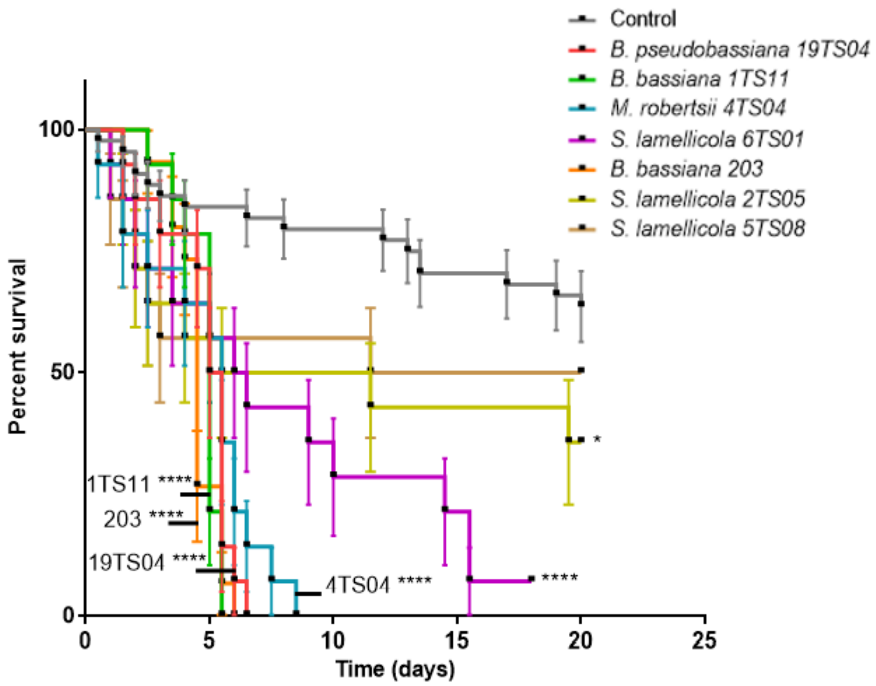

Figure S3. *B. pseudobassiana* 19TS04, *B. bassiana* 1TS11, *M. robertsii* 4TS04, *B. bassiana* 203 and *S. lamellicola* 6TS01 are significantly virulent on *G. mellonela* larvae at room humidity conditions. Asterisks indicate significant differences (*p<0.05, **p<0.01, ***p<0.001 and ****p<0.0001) respect to the control.

Table S1. Soil collection from banana fields in the Canary Islands. Abbreviations: Lat. = latitude, Long. = longitude, ASL = altitude, S = sprinkling, D = dripping, Cert. = certification, Int. P = integrated production, ECO = ecological crop, Conv. = conventional crop.

Code	Island	SIGPAC	Lat.	Long.	UTM	ASL	Irrigation	Cert.
**GS01**	La Gomera	38/21/16/9006/2	28°10'24'' N	17°11'17'' W	28 R 285181 3118341	82	S	No
**GS02**	La Gomera	38/21/15/13/2	28°10'38'' N	17°11'00'' W	28 R 285652 3118764	32	S	No
**GS03**	La Gomera	38/21/15/167/1	28°10'07'' N	17°11'31'' W	28 R 284790 3117824	83	S	No
**PS01**	La Palma	38/14/21/64/1	28°30'06'' N	17°52'16'' W	28 R 218959 3156139	56	S	Int. P
**PS02**	La Palma	38/7/6/306/1	28°49'49'' N	17°47'00'' W	28 R 228404 3192365	170	S	Conv.
**PS03**	La Palma	38/24/13/218/1	28°38'26'' N	17°55'09'' W	28 R 214628 3171650	267	S	ECO
**PS04**	La Palma	38/24/26/10/1	28°34'06'' N	17°53'36'' W	28 R 216961 3163582	69	S	Conv.
**PS05**	La Palma	38/24/26/82/1	28°33'40'' N	17°53'27'' W	28 R 217186 3162775	34	S	Conv.
**PS06**	La Palma	38/14/22/97/4	28°29'31'' N	17°52'20'' W	28 R 218824 3155063	10	S	Conv.
**PS07**	La Palma	38/30/7/156/1	28°44'58'' N	17°44'11'' W	28 R 232781 3183297	185	S	ECO
**PS08**	La Palma	38/24/13/56/1	28°38'31'' N	17°55'21'' W	28 R 214306 3171812	250	S	Conv.
**PS09**	La Palma	38/47/13/560/1	28°40'09'' N	17°56'08'' W	28 R 213103 3174861	360	S	Conv.
**PS10**	La Palma	38/30/13/252/1	28°46'44'' N	17°45'24'' W	28 R 230875 3186607	70	S	Conv.
**PS11**	La Palma	38/7/6/306/1	28°49'49'' N	17°46'58'' W	28 R 228459 3192364	160	S	Conv.
**PS12**	La Palma	38/14/10/36/2	28°29'08'' N	17°51'59'' W	28 R 219379 3154341	63	S	Conv.
**TS01**	Tenerife	38/26/3/48/ 1 a 6	28°24'23'' N	16°30'50'' W	28 R 351703 3143154	215	D	Int. P
**TS02**	Tenerife	38/31/9/181/2	28°23'28'' N	16°34'18'' W	28 R 346021 3141533	230	D	No
**TS03**	Tenerife	38/42/1/32/1	28°22'23'' N	16°48'51'' W	28 R 322228 3139866	50	D	Int. P
**TS04**	Tenerife	38/18/3/403/2 a 5	28°23'41'' N	16°40'12'' W	28 R 336391 3142063	115	D	Int. P
**TS05**	Tenerife	38/28/5/9065/9	28°24'00'' N	16°33'34'' W	28 R 347231 3142503	150	D	Int. P
**TS06**	Tenerife	38/10/5/17/3	28°21'53'' N	16°52'32'' W	28 R 316197 3139035	60	D	ECO
**TS07**	Tenerife	38/19/8/9000/136	28°10'48'' N	16°47'47'' W	28 R 323653 3118448	170	D	ECO
**TS08**	Tenerife	38/19/7/34/8	28°10'52'' N	16°48'02'' W	28 R 323245 3118577	135	D	Int. P
**TS09**	Tenerife	38/1/1/82/1	28°08'54'' N	16°47'43'' W	28 R 323710 3114937	70	D	Int. P
**TS10**	Tenerife	38/10/51/4	28°08'42'' N	16°47'16'' W	28 R 324441 3114557	80	D	Int. P
**TS11**	Tenerife	38/6/4/214/6	28°01'56'' N	16°38'58'' W	28 R 337858 3101869	75	D	Int. P
**TS12**	Tenerife	38/26/1/57/6	28°25'04'' N	16°30'43'' W	28 R 351910 3144413	100	D	Conv.
**TS13**	Tenerife	38/26/8/43/1	28°23'29'' N	16°32'30'' W	28 R 348961 3141526	91	D	Conv.
**TS14**	Tenerife	38/26/9/91/2	28°23'24'' N	16°33'04'' W	28 R 348033 3141384	252	D	Conv.
**TS15**	Tenerife	38/23/1/170/1	28°31'51'' N	16°23'47'' W	28 R 363375 3156803	75	D	Conv.
**TS16**	Tenerife	38/10/1/146/10	28°22'44'' N	16°50'20'' W	28 R 319815 3140550	95	D	Conv.
**TS17**	Tenerife	38/42/7/6/16	28°22'04'' N	16°48'13'' W	28 R 323254 3139266	72	D	ECO
**TS18**	Tenerife	38/23/2/44/3	28°31'38'' N	16°23'09'' W	28 R 364403 3156391	115	D	Conv.
**TS19**	Tenerife	38/26/1/29/1	28°24'36'' N	16°30'41'' W	28 R 351953 3143551	205	D	Conv.
**TS20**	Tenerife	38/26/2/13/1	28°24'38'' N	16°30'52'' W	28 R 351655 3143616	175	D	Conv.
**TS21**	Tenerife	38/19/5/233/1	28°11'36'' N	16°49'02'' W	28 R 321629 3119956	60	D	Int. P
**TS22**	Tenerife	38/26/3/50/2	28°24'37'' N	16°31'02'' W	28 R 351382 3143589	170	D	Int. P
**TS23**	Tenerife	38/19/2/939/1	28°12'51'' N	16°49'57'' W	28 R 320164 3122287	30	D	Int. P
**CS01**	Gran Canaria	35/09/2/111	28°09'28'' N	15°39'01'' W	28 R 436156 3114851	75	D	Conv.

**CS02**	Gran Canaria	35/6/6/1/1	28°08'05'' N	15°31'36'' W	28 R 448281 3112238	145	D	Conv.
**CS03**	Gran Canaria	35/9/2/103/2	28°09'27'' N	15°39'11'' W	28 R 435883 3114822	50	D	Conv.
**CS04**	Gran Canaria	35/6/6/10/1	28°08'01'' N	15°31'14'' W	28 R 448881 3112113	180	D	Conv.
**CS05**	Gran Canaria	35/6/8/272/1	28°08'24'' N	15°31'03'' W	28 R 449184 3112819	135	D	Conv.
**CS06**	Gran Canaria	35/6/8/455/3	28°08'10'' N	15°31'23'' W	28 R 448636 3112391	135	D	Conv.
**CS07**	Gran Canaria	35/9/1/244/1	28°09'11'' N	15°39'58'' W	28 R 434598 3114337	17	D	Conv.
**CS08**	Gran Canaria	35/6/7/312/1	28°08'14'' N	15°31'36'' W	28 R 448282 3112515	135	D	Conv.
**CS09**	Gran Canaria	35/9/1/260/4	28°08'49'' N	15°40'07'' W	28 R 434349 3113661	32	D	Conv.
**CS10**	Gran Canaria	35/9/1/288/1	28°09'03'' N	15°39'52'' W	28 R 434761 3114090	40	D	Conv.

Table S2. Fungal volatile organic compounds selected for the olfactometry bioassays.

Num.	Compound	Ret. Time (min)	Peak Height 10d	Peak Height 20d	Peak Height 30d	Peak Height 40d	Peak Height 50d	Peak Height 60d	Fungal species
**C1**	**Styrene**	18.55	-	-	-	-	-	-	Bb1TS11 Bb203
**C2**	**Benzothiazole**	31,00	NA	NA	23152	NA	NA	NA	Bb1TS11 (Bb203)
**C3**	**Camphor**	29.11	-	-	-	-	-	-	Bb1TS11
**C4**	**Borneol**	29,30	96273	24965	NA	31486	20551	NA	Bb1TS11
**C4**	**Borneol**	29,30	52467	48687	39535	39445	66449	40861	Bb203
**C5**	**1,3-dimethoxy-benzene**	28,75	263989	NA	NA	NA	NA	NA	Ma4TS04
**C5**	**1,3-dimethoxy-benzene**	28,75	7233098	509769	3583189	3807170	3046943	308330	Pc123
**C6**	**1-octen-3-ol**	22,36	26983	385375	141833	28896	NA	NA	Ma4TS04
**C6**	**1-octen-3-ol**	22,36	123365	108363	557934	NA	NA	308377	Pc123
**C7**	**3-cyclohepten-1-one**	15,20	50086	475717	306136	120587	NA	233415	Bb1TS11
**C7**	**3-cyclohepten-1-one**	15,20	274698	246124	103529	NA	NA	NA	Ma4TS04
**C7**	**3-cyclohepten-1-one**	15,20	714704	1027453	NA	NA	NA	NA	Pc123
**C7**	**3-cyclohepten-1-one**	15,20	179843	704724	147859	330871	239657	263098	Bb203

Table S3. Volatile organic compounds produced by the fungi analysed, in all the times measured. The list is made with a selection of compounds with a match percentage on the databases of at least 50%, without limitations in abundance.

Compound number	Compound name	Retention time (min)	Fungi
**1**	2-butanone	6,95	Pc123
**2**	Methyl ethyl, 2-butanone	6,99	Pc123
**3**	3-methylbutanal	9,10	Pc123
**4**	2-methylbutanal	9,43	Pc123
**5**	Dimethyldisulfide	12,60	Pc123
**6**	3-hydroxy-2-butanone	12,76	Pc123
**7**	4-methyl-2-pentanone	13,04	Pc123
**8**	3-methyl-1-butanol	13,28	Ma4TS04
**9**	2-chloro-octane	13,45	Ma4TS04
**10**	3-methyl-2-pentanone	13,46	Pc123
**11**	Cis-1-butyl-2-methylcyclopropane	13,49	Bb1TS11
**12**	1-octene	13,50	Bb203; Bb1TS11
**13**	1,3-octadiene	15,20	Ma4TS04; Bb1TS11
**14**	3-cyclohepten-1-one	15,20	Ma4TS04; Pc123; Bb203; Bb1TS11
**15**	Methyl-1,4-dioxide-pyrazine	15,24	Bb1TS11
**16**	(1-methylethenyl) cyclopropene	15,31	Pc123
**17**	3-hydroxy-2-pentanone	16,41	Pc123
**18**	Diethylamine-D1	16,41	Pc123
**19**	2,4-octadiene	16,64	Pc123; Bb203
**20**	4-methyl-3-hexanone	16,90	Pc123
**21**	Nonane	17,17	Pc123
**22**	3-methyl-3-penten-2-one	17,18	Pc123
**23**	5-methyl-2-hexanone	17,83	Pc123
**24**	α-pinene	19,40	Pc123; Bb203
**25**	2-butoxy-ethanol	19,79	Pc123
**26**	Anisole	19,80	Pc123
**27**	Methoxybenzene	19,82	Pc123
**28**	Propionoin	20,01	Pc123
**29**	2,4-dimethyl-3-hexene	20,54	Pc123
**30**	3,4-dimethyl-2-hexene	20,55	Pc123
**31**	Trans-3,4-dimethyl-2-hexene	20,55	Pc123
**32**	3,5-dimethyl-2-propylthiophene	20,61	Ma4TS04
**33**	β-pinene	21,20	Pc123; Bb203
**34**	1-β-pinene	21,24	Ma4TS04

**35**	2,2,4,6,6-pentamethyl-heptane	21,31	Bb1TS11
**36**	2-isopropyl-5-oxohexanal	21,42	Pc123
**37**	6-methyl-2-heptanone	21,42-25,13	Pc123
**38**	Propyl-cyclohexane	21,69	Pc123
**39**	Dimethyl-trisulfide	21,79	Pc123
**40**	(2R*,6R*,8AS*)-6-hydroxyedulan	22,01	Bb203
**41**	2-methyl-propanamide	22,14	Pc123
**42**	1-octen-3-ol	22,36	Ma4TS04; Pc123
**43**	3-octanone	22,40	Pc123; Bb203
**44**	3-methoxy-benzenamine	22,56	Ma4TS04
**45**	Trimethyl-pyrazine	22,70	Pc123
**46**	2-methyl-2-bornene	22,71	Pc123; Bb203; Bb1TS11
**47**	2,4-dimethyl-methyl ester-hexanoic acid	22,95	Pc123
**48**	6-methyl-bicyclo[3.3.0]oct-2-en-7-one	23,04	Bb203
**49**	1-methyl-4-(1-methylethyl)-benzene	23,18	Ma4TS04; Pc123
**50**	Isocyano-benzene	23,58	Pc123
**51**	2-(2-ethoxyethoxy)-ethanol	23,60	Ma4TS04; Pc123; Bb203; Bb1TS11
**52**	2,2,4,4,6,8,8-heptamethyl-nonane	24,26	Bb1TS11
**53**	2,4-dimethylfuran	24,41	Pc123
**54**	1,1'-oxybis-heptane	24,77	Bb203
**55**	2-dodecanone	24,9-34,28	Pc123
**56**	2-methyl-2-(2-methyl-2-butenyl)-furan	25,47	Pc123
**57**	Benzenemethanol	25,49	Pc123
**58**	(2-methyl-1-propenyl)-benzene	25,57	Ma4TS04
**59**	1-methyl-1H-1,2,4-triazole	25,58	Bb1TS11
**60**	2,3,3-trimethyl-1-butene	25,58	Bb203
**61**	2,2-dimethyl-propanal	25,63	Ma4TS04
**62**	2-methyl-3,4-dihydro-2H-pyran	25,70	Bb1TS11
**63**	6-methyl-4 5-dihydro.alpha.[2H]-pyran	25,70	Pc123
**64**	6,7-dimethoxy-2,2-dimethyl-2H-1-benzopyran	26,41	Pc123
**65**	2-amino-3,5-dibromo-6-methylpyridine	26,49	Ma4TS04
**66**	3-hydroxymandelic acid ethyl ester di-TMS	26,49	Pc123
**67**	4-hydroxymandelic acid ethyl ester di-TMS	26,49	Ma4TS04; Pc123; Bb203
**68**	4-trimethylsilyl-9,9-dimethyl-9-silafluorene	26,49	Pc123
**69**	p-Trimethylsilyloxyphenyl-bis(trimethylsilyloxy)ethane	26,50	Bb203
**70**	1-methylallyl(cyclooctatetraene)titanium	27,22	Pc123; Bb1TS11
**71**	4-octen-3-ol	27,55	Ma4TS04
**72**	4-fluoro-1,2-xylene	27,90	Ma4TS04; Bb203
**73**	1-phenyl-2-propanone	28,22	Pc123

**74**	1,3-dimethoxy-benzene	28,75	Ma4TS04; Pc123
**75**	2-amino-4-methylpyrimidine	29,14	Pc123
**76**	Bicyclo[2.2.1]heptane-2-carboxylic acid	29,14	Pc123
**77**	Borneol	29,30	Bb203; Bb1TS11
**78**	9H-pyrrolo[3',4':3,4]pyrrolo[2,1-a] phthalazine-9,11(10H)-dione,10-ethyl-8-phenyl	30,54	Ma4TS04
**79**	1-(2-furanyl)-3-butene-1,2-diol	30,96	Ma4TS04
**80**	2,4-bis(1,1-dimethylethyl)-phenol	31,00	Ma4TS04; Pc123
**81**	Benzothiazole	31,00	Bb1TS11
**82**	2-undecanone	31,15-32,18	Pc123
**83**	16-oxosalutaridine	31,48	Pc123
**84**	5-oxoisoboldine	31,48	Ma4TS04
**85**	4,4-dimethyl-6-benzylamino-dihydro-8H-thiopyrano[4',3':4,5]thieno[2,3-d]pyrimidine	31,50	Ma4TS04; Pc123
**86**	2-octanone	32,19	Pc123
**87**	1-ethyl-2-methyl-cyclohexane	33,21	Pc123
**88**	5-ethyl-3-hydroxy-4-methyl-2(5H)-furanone	33,21	Bb1TS11
**89**	5-acetyl-2-hydrazino-4-methylpyridine	33,70	Pc123
**90**	β-elemene	34,61	Pc123
**91**	2-methoxy-3,8-dioxocephalotax-1-ene	34,86	Pc123
**92**	Di-amylcyclohexanol	34,97	Pc123
**93**	Cyclotetradecane	36,68	Pc123
**94**	(+-)-gymnomitrene	37,10	Ma4TS04; Bb1TS11
**95**	1,4-bis(methylene)-cyclohexane	37,10	Ma4TS04
**96**	2-tridecanone	37,60	Pc123
**97**	trans-β-farnesene	37,94	Pc123

References

1. Tamura K. and Nei M. (1993). Estimation of the number of nucleotide substitutions in the control region of mitochondrial DNA in humans and chimpanzees. Molecular Biology and Evolution 10:512-526.
2. Kumar S., Stecher G., Li M., Knyaz C., and Tamura K. (2018). MEGA X: Molecular Evolutionary Genetics Analysis across computing platforms. Molecular Biology and Evolution 35:1547-1549.
